# Evaluation and Development of Analytical Procedures to Assess Buffering Capacity of Carbonate Ruminant Feed Buffers

**DOI:** 10.3390/ani14162333

**Published:** 2024-08-13

**Authors:** Patrick Quille, Tommy Higgins, Enda W. Neville, Katy Regan, Shane O’Connell

**Affiliations:** 1 Shannon Applied Biotechnology Centre, Munster Technological University Kerry, Clash, V92CX88 Tralee, Ireland; patrick.quille@mtu.ie; 2 Marigot Researh Centre, Sycamore Court, Clash, V92 N6C8 Tralee, Ireland; 3 Celtic Sea Minerals, Strand Farm, Currabinny, P43 NN62 Carrigaline, Ireland

**Keywords:** rumen acidosis, sub-acute rumen acidosis, dairy cows, rumen buffer, calcareous marine algae, rumen pH, titration

## Abstract

**Simple Summary:**

Rumen buffers are included in ruminant diets to prevent the accumulation of excess acid in the rumen, which can lead to animal health issues and production losses. Laboratory methods to compare different rumen buffers are useful to select the optimal buffer for inclusion in ruminant diets. The influence of key method parameters such as buffer material, threshold pH and test duration are reported. The area under the titration curve was identified as the most sensitive measure of buffer performance. Current laboratory methods to assess the efficacy of rumen buffers do not correlate very well with actual rumen measurements. A new method to model an acidotic rumen provided results that were better related to in vivo data.

**Abstract:**

The inclusion of rumen buffers in ruminant feeds has gained widespread adoption for the prevention of rumen acidosis, thereby avoiding the negative production and health consequences of low rumen pH and resulting in improved feed efficiency. Benchmarking and quality controlling the performance of rumen buffer materials is of significant interest to feed mills and end-user producers. The aim of this study was to evaluate, develop and optimise a laboratory protocol to consistently and robustly evaluate rumen buffering materials in order to predict their in vivo efficacy. Three different methods were evaluated for determining the buffering potential of carbonate buffer materials: (a) 2 and 8 h static pH, (b) 8 h fixed HCl acid load addition and (c) 3 h acidotic diet simulation using acetic acid. Buffer material, threshold pH, test duration and interactions between all three variables were significant (*p* < 0.001) in evaluating the performance of the buffer materials. The acidotic diet simulation was found to provide a different ranking of materials to the 8 h fixed HCl acid load methodology. The results highlight the importance of method selection and test parameters for accurately evaluating the potential efficacy of rumen buffer materials.

## 1. Introduction

Ruminant diet formulation is essential when it comes to animal productivity. In order to fulfil the energy requirements for a productive dairy cow, highly fermentable diets are commonly fed [1]. These highly fermentable diets are often high in starch and limited in the amount of effective fibre they contain, which may alter the volatile fatty acid (VFA) levels in the rumen [2]. VFAs, including lactic acid, are produced during the fermentation of feedstuff in the rumen; however, accumulation of these acids may lead to a drop in rumen pH [3]. Increased rumen acidity can result in metabolic disorders, with sub-acute rumen acidosis (SARA) being one of the most common [1,4]. SARA is of concern amongst dairy farmers, as it reduces cow productivity, increases the risk of adverse health conditions [2,5,6] and can lead to the animal’s death [1]. The definition of SARA is a depression in rumen pH for more than 3 consecutive hours per day below a pH of 5.6 [7]. To maintain a healthy rumen pH and reduce the risk of rumen acidosis, dietary alterations must be put in place [8,9]. The rumen pH depends on the Henderson–Hasselbalch equilibrium (which describes buffering and acid–base balance) and is given by the equation pH = pK_a_ + log ([A^−^]/[HA]); ([HA] and [A^−^] refer to the equilibrium concentrations of the conjugate acid–base pair), where pK_a_ is the negative log of the acid constant, K_a_. Lactic acid has a lower pK_a_ (3.86) than acetic acid (pK_a_ = 4.76), propionic acid (pK_a_ = 4.87) or butyric acid (pK_a_ = 4.82) [10], meaning it is a stronger acid; therefore, rumen pH is more affected by the production of lactic acid. Thirty percent of VFAs are neutralized by salivary sodium bicarbonate (SB) and phosphate, whose production is stimulated by chewing and the amount of effective dietary fibre [5,11]. If the capacity of the endogenous buffer system is exceeded due to a highly fermentable diet with low fibre content, the production of VFA can exceed removal via the rumen wall, resulting in a drop in rumen pH [12]. The lower pH favours the growth of lactic acid-producing bacteria in the rumen, and the increased accumulation of low pK_a_ lactic acid results in a shift in VFA profiles and a decrease in pH occurs, resulting in acute acidosis [12].

The inclusion of rumen buffers to the ruminant diet is one intervention that can be implemented to regulate rumen pH [13,14] and suppress SARA in dairy cows [4,15] while simultaneously combating milk fat depression [16]. Dietary buffers reduce rumen acidity and create a more desirable environment for microbial activity [17,18]. This can lead to enhanced rumen microbial growth, increased enzymatic activity and higher microbial diversity and thus contribute to improved fermentation efficiency and nutrient availability [19]. Rumen buffers are typically composed of mineral salts and calcareous marine algae (CMA) products [14,16,20].

Sodium Bicarbonate (SB) is widely used as a rumen buffer [13,21,22,23,24]. SB buffering action is short lived as a fully soluble buffer in the rumen and cannot buffer ongoing acid production [15,23]. Furthermore, Cruywagen et al. reported CMA (*Lithothamnion* sp.) had a higher buffering capacity and higher minimum rumen pH when compared to (SB) when fed to dairy cows over the course of sixty-six days [20]. An in vivo study conducted by Neville et al. explored the effect of different rumen buffers on rumen pH and milk production in mid-lactation dairy cows fed a high-starch TMR based on ryegrass silage and corn silage [15]. The study reported differences in in vivo rumen pH, milk production and milk quality depending on the type of buffer fed. Similar data are available highlighting the milk production benefits of CMA when fed to pasture grazing dairy cows [25]. The influence of rumen buffers on in vivo dairy cow productivity has therefore been well reported.

However, laboratory-based rumen buffer testing protocols can produce results that are contradictory to the reported in vivo performance. Historic laboratory testing protocols were based on total acid neutralisation capacity of feed and/or rumen buffers [10,26,27]. However, these methods do not take into account the rate of acid production in the rumen or the actual rate of change of pH for different rumen buffer materials over a fixed period of time. This study aims to provide an in-depth evaluation of analytical protocols to calculate the buffering capacity of commercially available rumen buffer materials (e.g., CMA, calcium carbonate and SB), along with assessing how their composition and particle size might affect buffering performance, in order to determine the analytical factors that affect the prediction of buffering capacity and their role in developing an in vitro protocol that correlates better with in vivo performance.

## 2. Materials and Methods

### 2.1. Rumen Buffer Materials

Commercial CMA materials are currently available as feed additives/rumen buffers and include Lithothamnion Calcareum 1 (CMA Cal 1), Lithothamnion Calcareum 2 (CMA Cal 2), Lithothamnion Calcareum 3 (CMA Cal 3) and Lithothamnion Glaciale (CMA Glac). These buffer materials were provided by Celtic Sea Minerals (Strand Farm, Currabinny, Carrigaline, Co. Cork, Ireland). Feed-grade limestone/calcium carbonate (Calc Carb) and sodium bicarbonate (SB) materials were obtained from commercial sources in the European market.

### 2.2. Compositional and Physical Characterisation of Calcium Carbonate Buffer Materials

The inorganic and organic composition of the carbonate materials was determined by heating 3 g of material in triplicate in a furnace at 450 °C for 5 h. Samples were placed in a desiccator to cool overnight and were subsequently weighed. The percentage inorganic matter was calculated by dividing the ash weight by the starting weight of the material and multiplying by 100. The organic matter was calculated by the difference between the material weight and the ash weight. The calcium and magnesium composition was determined by first digesting 200 mg of the carbonate materials in 10 mL of nitric acid (65%) in a microwave digester (Mars 6, 240/50 CEM Microwave Technology (Ireland) Ltd., Dublin, Ireland) ramped to 190 °C over 20 min, held at 190 °C for 15 min, cooled for 20 min to 70–80 °C and subsequently analysed using a suitable dilution on an Agilent ICP-MS 7800 instrument (Agilent Technologies Ireland Ltd., Cork, Ireland) tuned using Agilent Tuning Solution for ICP-MS (1 ppb), part No. 5185–5959, and calibrated with Agilent Environmental Calibration Standard, part No. 5183–4688. All ICP-MS elemental calibration curves had r-squared values >0.95.

The particle size distribution was performed using a Mastersizer 3000 (Malvern Panalytical, Malvern, UK). A representative sample (1 g) of each material was added to the sample port of the Mastersizer 3000, which calculated the particle size via software that measures the intensity of light scattered as a laser beam passes through a sample particulate flowing through the instrument; all analysis were performed in triplicate.

#### 2.2.1. Static pH Titration for Acid Binding and Buffer Capacity Measurement

The pH stat methodology was used to evaluate the acid binding capacity of buffer materials at rumen relevant pH values of 5.5 (threshold for SARA), 5.8 (threshold for sub-optimal rumen conditions) and 6.0 (optimal). The acid binding capacity was determined using a Titrando automated potentiometric titrator (Metrohm Ltd., Herisau, Switzerland) and was based on the method reported by Rafferty et al. [25]. In brief, the Titrando was calibrated utilising a pH calibration programme and calibrator fluids (Scharlab, Barcelona, Spain) at pH 2.0, 4.0, 7.0 and 9.0. A calibration slope of 0.99 was required for the calibration to be acceptable. The buffer materials were evaluated by weighing out 0.25 g, which was subsequently added to 150 mL of water (0.1 μS/cm Type 2) in a 250 mL beaker. The potentiometric probe and the acid dispenser of the Titrando were subsequently inserted into the sample beaker, stirring at a rate of 1000 rpm, with a cylindrical stirrer bar with dimensions L = 24 × D = 4 mm; the titration was performed at 19–21 °C. The static pH programme was selected through the Titrando software. The programme titrated the sample with 0.1 M HCl (Honeywell Fluka™, Charlotte, NC, USA) to the first end point of pH 7 (the molarity of HCl was confirmed by titration with 1.0 M NaOH using phenolphthalein as an indicator). The volume of 0.1 M HCl was recorded by the programme. The programme then titrated the sample against 0.1 M HCl to the second end point of the target static pH and held the pH for the duration of the titration. The Titrando software recorded the total volume of HCl required to achieve and maintain the set pH values. The moles of H^+^ used were calculated from the volume and concentration of HCl. The uncertainty of measurement (UoM, u) was calculated by first calculating the standard error of mean (SEM) of the within-day precision (A) and the standard deviation of the between-day precision (B). Once calculated, both A and B were squared and added together, with a final calculation of the square root (u = √ A^2^ + B^2^). The acid binding capacity (ABC) was determined using the following equation: ABC at target pH in meq/Kg = mL HCl titrated × Molarity of HCl × 4000. The buffer capacity (BUF) was calculated by the following equation: BUF of target pH = ABC/(Initial pH − final pH) as previously outlined by Lawler et al. [28].

#### 2.2.2. Fixed HCl Acid Load Titration

The fixed HCl acid load titration was performed by weighing out 0.2 g of buffer material into a 250 mL glass beaker. A total of 150 mL of 0.1 μS/cm Type 2 De-ionised Water (Wasserlab Autowomatic, Navarra, Spain) was added to 0.2 g of product, with the cylindrical stirrer bar added just before starting the Titrando programme. The Titrando programme continuously added 200 μL of 1.0 M HCl every 25 min over a period of 800 min. The Titrando continuously monitors and records the pH value of the test solution every 10 s over the entire duration of the programme.

#### 2.2.3. Calculation of Rate of Change of pH (dpH/dt) and Area under the Curve (mmol H^+^.s)

The fixed acid load titration method measures the pH oscillations from a defined volume of acid added every 25 min over a 13 h period. The programmed method enables a continuous pH measurement throughout the duration, which results in a potentiometric graph of pH versus time. The data obtained from the potentiometric titration allow for the evaluation of kinetic parameters of the pH change over time. Using the 1st derivative, the rate of change of pH over time (dpH/dt) was calculated and averaged for the duration that the pH remained above optimal (pH 6.0), sub-optimal (pH 5.8) and SARA (pH 5.5) pH values for each buffer material tested (the rate of change of pH over time would reflect the buffering ability of each material to maintain a steady pH over time). An additional parameter that was calculated was area under the curve (AUC = mmol H^+^.s is a measure of mmol of acid neutralized over a threshold pH over a defined period of time). This parameter was calculated in intervals of 10 s by using Riemanns numbers [29] to calculate the area between the measured pH curve obtained from the fixed acid load titration, the threshold pH and upper normal rumen pH of 7.5 [15]. The area was calculated by converting the positive difference between threshold pH (6.0 optimal, 5.8 sub-optimal or 5.5 SARA) and the measured pH (within the maximum pH of 7.5) at 10 s intervals to mmol H^+^ using the Henderson Hasselbalch equation ($pH={pK}_{a}+log\times \frac{\left[base\right]}{\left[acid\right]}$). Subsequently, each calculated area expressed as mmol H^+^.s was summed to provide an AUC expressed as mmol H^+^.s for each material.

#### 2.2.4. Determination of Area under the Curve for In Vivo Rumen Acidosis Trial

An in vivo trial evaluating the effect of different buffer materials on rumen pH of dairy cows fed an acidotic diet was previous published by Neville et al. [15]. Briefly, rumen pH was measured using internal pH probes linked to a data logger (Intech Instruments Ltd., Lincoln, New Zealand). The pH data loggers were connected to straps that were securely fastened around the shoulder of the cow to prevent damage to the data logger while also avoiding irritation of the cow. The electrodes were housed in specially designed stainless-steel capsules and joined to the cannulas via water-tight hoses and fittings. This specially designed rumen cannula, holding the pH probe, allowed the pH probe to reside in the centre of the rumen. On day 2 of each data collection period, the pH loggers and probes were introduced at 11:00 h, 2 h after feeding. The pH probes were cleaned, checked for accuracy, and re-calibrated, with pH 4.0 and 7.0 standards every 24 h. Continuous pH measurements from the indwelling probe were sent to the data logger every 10 min. After the device had been removed each day, pH measurements were retrieved from the data logger. Measurements taken over the 3 days were combined. pH measurement data from the trial were processed to calculate AUC as outlined in Section 2.2.3.

#### 2.2.5. Three-Hour Acidotic Diet Simulation Using Acetic Acid

The in vitro acidotic diet simulation aimed to create an in vitro simulation of the rumen pH environment, which was informed by in vivo rumen pH and VFA concentration data obtained from cows experiencing SARA, as outlined in Neville et al. [15]. The concentration of acetic acid and addition rate was calculated based on the VFA and pH profiles measured in the in vivo rumen environment. A total of 150 mL of acetic acid solution (67 mM; Honeywell Fluka™) was prepared in a 250 mL beaker and adjusted to an acidotic rumen pH (5.8) with the addition of 1.0 M SB (Honeywell Fluka™). Then, 0.1 g of the buffer products was added to the 150 mL solution of acetic acid (Honeywell Fluka™) and SB. The beaker was sealed with parafilm (Bemis Co. Lab, Buckinghamshire, UK) to retain the generated CO_2_ and to create a semi-permeable closed system. The rate of acid addition was determined from the pH profile reported in Neville et al. [14] using the Henderson–Hasselbalch equation ($pH={pK}_{a}+log\frac{\left[base\right]}{\left[acid\right]}$); the free acid left in the buffer system was calculated in order to determine the rate of acid addition over time to achieve the same rate of pH decrease as measured in vivo. The buffer products were titrated against 1.0 M acetic acid (Honeywell Fluka™) using the Titrando, which was programmed to continuously add 0.35 mL every 25 min for a duration of 180 min (the rate of addition of acetic acid was calculated from the in vivo pH profile and VFA concentration data; see supplementary data Figures S1 and S2). The pH was recorded every 10 s, resulting in a graph of pH versus time. The AUC was calculated as outlined in Section 2.2.3.

#### 2.2.6. Statistical Analysis

The data are expressed as mean ± standard error of at least three independent experiments, with actual replicates indicated in figures and tables. All data were analysed using the XLSTAT software package version 2014.5.03. Where appropriate, data were analysed using either one-way analysis of variance (ANOVA) for one variable, a two/three-way ANOVA (for comparing multiple factors with interactions) or a Kruskal–Wallis non-parametric test for comparison of multiple experimental variables that were not normally distributed, following a Grubbs test for outliers and determination of normal distribution of data using the Shapiro–Wilk test. Tukey’s post hoc test was used for variable group comparisons to test for significant differences between variable means. Differences were significant at *p* < 0.05.

## 3. Results

### 3.1. Compositional and Physical Characterisation of Carbonate Based Buffering Materials

The calcium carbonate based materials used in this study were evaluated for compositional (Table 1) and physical parameters (Table 2), which have been reported to have a role in their buffering properties. It is evident in Table 1 that there are sizeable and significant differences between buffer materials in % organic matter, calcium and magnesium content. The organic content varied from 0.45 to 3.63% *w*/*w*, and the magnesium content ranged from 0.13 to 5.67% *w*/*w*.

It is apparent in Table 2 that there are significant differences between materials in terms of their particle distribution; however, the material with the maximum particle size (CMA Cal 1), while significantly different to the others, is not of a large magnitude (Dx 99 showing less than 6% between the biggest and smallest; CMA Cal 1 vs. CMA Cal 3). However, the distributions between the materials are statistically different, with the two most similar distributions being CMA Glac and CMA Cal 2.

### 3.2. Static pH Acid Binding and Buffer Capacity

Data estimating the precision and uncertainty of measurement (UoM) of the static pH titration methodology used in this study are presented in Table 3. The coefficient of variation ranges between 1.1 and 28.7%, with the buffer material having an influence on the level of variation. The UoM ranged between 0.51 and 4.39 mL of HCl. The between-day and within-day variation does not appear to be consistent for both static pH value and buffers.

The results presented in Table 4 for acid binding capacity (ABC) and buffering capacity (BUF) for the different buffer materials evaluated in this study show significant differences in ABC and BUF values, which range from 24,127–5296 Meq/Kg and 9060–1285 Meq/pH unit, respectively. Significant differences between the durations of the test were evident for some of the buffer material, such as CMA Glac, where the ABC value increased from 11,194 Meq/Kg for the 2 h duration to 17,979 Meq/Kg for the 8 h duration. SB had a minor but not significant increase in ABC from 2 h to 8 h.

CMA Glac had 111% higher ABC than the lowest comparator CMA, CMA Cal 1 at pH 5.5 over 2 h. Similar trends were also evident from the calculated BUF values. The coefficient of variation for the measured ABC and BUF values were higher for some of the tested samples at 2 h in comparison to the 8 h duration.

### 3.3. Fixed HCl Acid Load Titrations

An example of the potentiometric titration curves from the fixed acid load titration of the buffer materials is presented in Figure S3. These titration curves were produced in triplicate for each material, and the data were subsequently used to calculate the parameters presented in Table 5, as previously outlined.

Table 5 provides data on the statistical significance and magnitude of the effects of the key variables identified in the fixed HCl acid load titration methodology. All the variables identified had a significant effect on AUC. Buffer material and duration of the titration had a significant effect on dpH/dt, and time pH was greater than threshold pH. The mean AUC of SB at double the concentration over all levels of the variables had the highest AUC (34.13 mmol H^+^.s), with significant differences evident between CMA Glac (24.37 mmol H^+^.s) and the other CMAs and calcium carbonate (7.73–22.08 mmol H^+^.s). There were significant interactions between all three variables for AUC. The buffering rate was expressed as the average dpH/dt over a 2 h and 8 h duration, with an average of the rate of change being calculated up to the point the pH remained above the test threshold pH. SB recorded the lowest rate of change, 1.84, expressed as the average change in pH units over the test duration. CMA Cal 3 recorded lower reaction rates (4.74) compared to the other CMA buffers and calcium carbonate (*p* ≤ 0.05). Variable duration and buffer material had a significant effect on dpH/dt, with an interaction between the variables also being significant.

**Figure 1:**
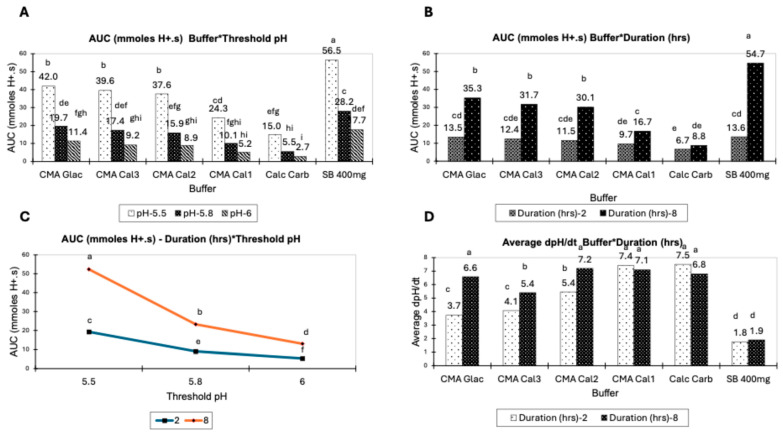
Interactions between variables of the fixed HCl acid load titration methodology. (**A**) Interaction between buffer material and method threshold pH for calculated AUC, (**B**) interaction between buffer material and test duration for calculated AUC, (**C**) interaction between test duration and threshold pH for calculated AUC, (**D**) interaction between buffer material and duration for calculated dpH/dt. Data expressed as means ± SE. n = 5; pairwise comparison completed using Tukey’s post hoc test, *p* < 0.05; different superscript letters denote statistically significant differences.

The interactions between method variables with statistical significance are presented in Figure 1. It is evident from the data that the threshold pH used in the test methodology has a significant effect on the AUC for all buffers tested (Figure 1A). The data also indicate a significant effect for test duration on the AUC for all buffers (Figure 1B); however, the magnitude of the effect is variable and related to the buffer being tested. The interaction between test duration and threshold pH (Figure 1C) was significantly different for all tested combinations, which indicates that the selection of the levels of these variables and keeping them constant are important for the accurate comparison of buffers.

The only significant interaction for dpH/dt was between buffer and test duration (Figure 1D). This indicates that as the buffer is being depleted, the control of pH becomes more erratic for some materials, but for the soluble SB this does not occur.

### 3.4. Three-Hour Acidotic Diet Simulation Using Acetic Acid

The results of the evaluation of the buffer materials using the 3 h acidotic diet simulation are presented in Table 6, with CMA Glac (9.58 mmol H^+^.s) and 2x SB (9.85 mmol H^+^.s) being the two most efficient materials and CMA Cal 1 being the poorest performing (5.73 mmol H^+^.s). The table includes the statistical evaluation of the impact of the variables of the test that are relevant to in vivo rumen buffering, such as degree of acidosis, represented by threshold pH and the duration of the buffering effect. All the test variables evaluated had a significant effect on AUC (*p* < 0.001), while dpH/dt was significant for buffer material and duration of the test. Significant two-way interactions were also found for AUC.

**Figure 2:**
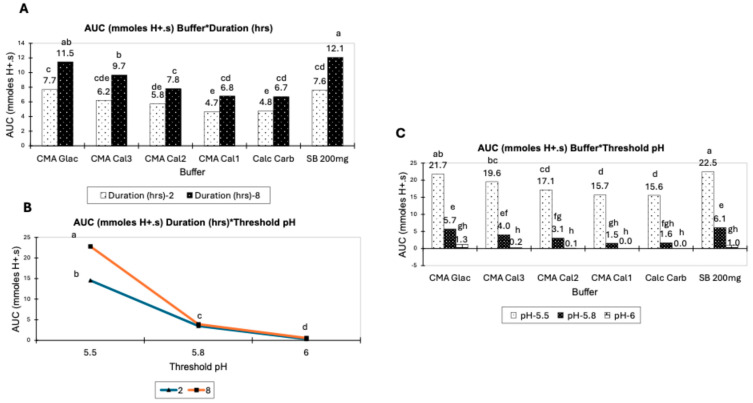
Interactions between variables of the acidotic diet simulation methodology. (**A**) Interaction between buffer material and method threshold pH for calculated AUC, (**B**) interaction between buffer material and test duration for calculated AUC, (**C**) interaction between test duration and threshold pH for calculated AUC. Data expressed as means ± SE. n = 5; pairwise comparison completed using Tukey’s post hoc test, *p* < 0.05. Different superscript letters denote statistically significant differences.

The interactions between method variables for the acidotic diet simulation with statistical significance are presented in Figure 2. The threshold pH used in the test methodology again had a significant effect on the AUC for all buffers tested (Figure 2A). The data also indicate a significant effect for test duration on the AUC for all buffers (Figure 2B). The interaction between test duration and threshold pH (Figure 2C) was significantly different at threshold pH 5.5 only.

The AUC from the in vivo trial evaluating the impact of buffers in a diet-induced acidosis is presented in Table 7. The AUC was identified as being the most informative variable in the in vitro simulation based on its significance in being influenced by test variables time, threshold pH and buffer material. Therefore AUC was used to evaluate the in vivo acidosis trial data.

There were significant differences between the control diet using limestone (9.65 mmol H^+^.s) and the CMA Glac and SB diet (14.1 mmol H^+^.s and 14.3 mmol H^+^.s, respectively).

## 4. Discussion

The focus of this study was to evaluate and optimise laboratory protocols to consistently and robustly evaluate carbonate-based rumen buffering materials in order to provide benchmarking and prediction of their in vivo efficacy and ultimately animal performance, through more efficient rumen pH management. Compositional and physical characterisation of the different calcium carbonate materials was performed in order to assess the variation between the materials and to assess the contribution of this variation to efficacy. It is evident from the data presented in Table 1 and Table 2 that significant variation exists between individual CMA materials and calcium carbonate for the tested parameters. This variation is particularly apparent in the organic matter, calcium and magnesium content. All four CMA materials have statistically significant differences in organic matter, with CMA Cal 2 and CMA Glac having the highest content. The presence of organic matter in CMA has been previously reported. Components of this organic matter have been described to have a key role in the biomineralisation process of red seaweed during its vegetative growth phase [30]. A number of different species of CMA exist, with the distribution of species being specific to geographical location, water temperature, depth of water and ocean currents [31]. Differences between the organic, calcium and magnesium content have been reported for different CMA species [30,32]. The variation in compositional data reported in this study likely reflects the use of different CMA species for the manufacture of rumen buffers. The composition of calcium carbonate is in agreement with previously reported values. The particle size distribution of the materials is similar, but there are statistically significant differences in the distribution. The differences in the particle size is likely associated with the processing of the materials rather than material-specific characteristics. The relationship between acid neutralisation capacity and calcium carbonate-based particle size has been reported [33], with a range of particle size from 37.7 to 3067.9 µm for limestone [33].

Three different methods were evaluated for determining the buffering potential of rumen buffer materials: (a) 2 and 8 h static pH, (b) 8 h fixed HCl acid load addition and (c) 3 h acidotic diet simulation using acetic acid. All methods are based on acid–base titration, with the 2 h static pH based on previously published methods for buffer capacity (BC) and buffer value index (BVI) methods, with modification to focus on evaluation of the buffers only over a duration rather than total diets and/or rumen fluid [27,34]. The uncertainty of measurement (UoM) of the static pH titration methodology was determined at pH 5.5 and 6.0 and was found to range from 0.51–4.39 and 0.93–2.55 mL of HCl, respectively (Table 3), for five different carbonate buffer materials. Data on UoM for feed buffer evaluation were not found in the published literature for comparison. Calcium carbonate had the highest UoM (2.55–4.39), which is likely due to inherent variability in the material, which has been widely reported for limestones of various origins [33,35,36]. All the other materials had similar UoMs, which indicates the variation of the titration methodology was between 0.51 and 2.1 mL of 0.1 M HCl when performed as outlined in this study. Similar coefficients of variation (1–8%) have been reported in other studies for solubilisation and neutralisation of calcium carbonate-based materials [37,38].

Other laboratory testing protocols for assessing rumen buffering capacity are based on total acid neutralisation capacity or acid binding capacity (ABC) of feed and/or rumen buffers [10,26,27]. The results presented in Table 4 show that the length of time of the titration (2 or 8 h) has a significant effect on ABC and BUF values for all materials, except SB, with values increasing in ABC and BUF over time, which is likely to be relevant in an in vivo rumen environment. A prolonged buffering action is desirable for dairy cows fed a highly fermentable diet to avoid periods of sub-optimal rumen pH; therefore, those materials having prolonged buffering action are likely to be beneficial in vivo [15]. There was no sizeable increase in ABC or BUF with SB over time, which is likely due to its high solubility and indicates that it has a quick acting buffering action, which has been observed in vivo [15]. In Table 4, the best performing buffers are SB and CMA Glac. CMA Glac has approximately 100% higher ABC than CMA Cal 1 and calcium carbonate at pH 5.5 over 2 and 8 h. This result highlights the significant differences between CMA buffers, which is likely associated with the species of CMA, their location of origin and their inherent composition. CMA Glac had significantly higher organic matter and magnesium content than CMA Cal 1 and CMA Cal 3, with the divalent basic forming cation, magnesium, of marine origin likely affecting the acid neutralisation capacity of CMA Glac. Processing of the CMA buffers and their particle size distribution are also likely play a role in the observed ABC values, as increased surface area is likely to enhance ABC. However, the significantly smaller particle size distribution in CMA Cal 3 did not provide values that were higher than CMA Glac, so other factors may be contributors, such as surface area from the inherent pore space in the material, which should be evaluated in the future. Similar trends are also evident from the calculated BUF values. Evaluation of the extended period of 8 h at pH 5.5 confirmed the trend observed at the same pH over 2 h, where the CMA Glac and SB materials provided the highest ABC and BUF values. It was clear in each method that 65–72% of SB was consumed to reach the first end point of pH 7, thus demonstrating the influence of the pK_a_ value of SB [34]. Additionally, the high solubility and pK_a_ value of SB limits the buffering capability of the material below pH 6, as the alkaline species of SB disassociates at pH 6.3 to form the intermediate carbonic acid, with up to 20% of the initial concentration converting to the intermediate and subsequently carbon dioxide at pH 6.0 [34]. The ABC of all the materials at pH 6 is lower than that reported in the literature for calcareous materials (calcium carbonate = 18,000–20,000 meq/Kg), but this is likely due to the target pH being pH 3–4, in line with monogastric nutrition requirements [28]. Previous evaluation of the performance of an SB buffer combined with a defined diet in rumen fluid was reported as BC with values of 70–85 meq/L of rumen fluid reported for the control with no buffer and 110–125 meq/L reported for SB [34]. However comparison of the BC and BVI values with ABC and BUF values as determined in this study is not possible due to different experimental setups and testing objectives, with BC and BVI focused on total feed or rumen contents.

ABC and BUF testing protocols can produce results that are contradictory to the reported in vivo performance [18,20]. These methods do not take into account the rate of acid production in the rumen or the actual rate of change of pH for different rumen buffer materials over a fixed period. The fixed HCl acid load titration was developed as an in vitro method to evaluate the performance of buffers using a methodology that has a closer relationship with the rate and dynamics of acid production in the rumen environment over time. The fixed HCl acid load methodology was evaluated to determine the method variables that have a significant effect on predicting a buffer potential to prevent suboptimal rumen pH and SARA (pH 5.8 and 5.5, respectively), reported as area under the curve (AUC, mmol H^+^.s), rate of change of pH over time and length of time pH, was greater than the threshold pH for SARA diagnosis. SB and CMA Glac were the two best performing buffer materials in this method, having the highest values for the three calculated variables of AUC, dpH/dt and time above threshold pH, which is in line with ABC and BUF values derived from the static pH methodology. Of the three calculated output measures from the titration curves, AUC was identified to be the most sensitive measure. Buffer material, threshold pH, test duration and interactions between all three variables significantly (*p* < 0.001) affected AUC values, and thus this output measure was selected to predict performance of the buffer materials. AUC had been previously shown in vivo to differentiate between pH profiles of low-fibre and high-fibre diets, which further supports it utilisation in an in vitro testing methodology [39].

HCl is a strong acid whose pKa (pKa = −8.0) is much lower than rumen relevant organic acids and therefore unlikely to accurately model rumen acid–base dynamics. Therefore, an in vitro test system that utilises a rumen relevant organic acid such as acetic acid (pKa = 4.76) is likely to be a better indicator of in vivo buffering performance [40]. pH and acid load are not static in the rumen; they are constantly changing based on the animal’s diet, eating pattern and production system. Therefore an estimation of the rate of acid addition from in vivo data on pH changes is likely to yield a better estimate of in-practice in vivo performance. The three-hour acidotic diet simulation using acetic acid developed in this work was optimised using data obtained from an in vivo trial using cannulated dairy cows [15]. The simulation was found to provide a different ranking of materials to the ABC/BUF values and the 8 h fixed HCl acid load methodology. The main difference in the comparison of the materials was the reduction in the predicted buffering capacity of SB in the acidotic diet simulation. The predicted reduced buffering capacity of SB is likely associated with its pKa (SB pKa = 6.3) [41]. A pKa of SB of 6.3 means that its buffering capacity will become limited at pH levels below 6.3. When SB is not in excess of the rumen acids and the rumen pH is less than the pK_a_, it starts to convert to dissolved carbon dioxide (dCO_2;_ with carbonic acid as an intermediate species) [37,41]. Additionally, Kohn and Dunlap describe the influence of the partial pressure of carbon dioxide (pCO_2_), where it increases in systems from which CO_2_ escape is held up and CO_2_ is forced back into solution, which results in lowering of the pH [10]. Experimental data to validate the build-up of dCO_2_ and the subsequent decrease in the rumen pH have been reported, with dCO_2_ recognised as being more dominant than VFAs in rumen acidosis [41]. The increase in dissolved CO_2_ has also been shown to be independent of the pCO_2_ and dependent on other rumen fluid factors, such as fluid viscosity, surface tension and temperature. Therefore, current research underestimates rumen dCO_2_ concentrations, because it assumes a linear relationship with pCO_2_ for indicating the concentration and because it employs in vitro conditions. However, liquid CO_2_ species are the main source of rumen CO_2_, and Henry’s law cannot predict dCO_2_ in a non-ideal rumen fluid [42].

The acidotic diet simulation highlights the limitations of SB as a rumen buffer, which are not demonstrated by the HCl-based methodologies but are correlated with in vivo data [15]. The AUC values from the in vivo data presented in Table 7 highlight the correlation of the acidotic diet simulation over an initial 3 h period after the first feeding period of the cows in the day. However there are sizeable differences between the calculated in vivo AUC (14.3 mmol H^+^.s) and in vitro AUC (6.63 mmol. H^+^.s) for SB, which are likely associated with the starting rumen pH (6.2 and 5.8, respectively).

## 5. Conclusions

The results reported in this study highlight the importance of method selection and test parameters for accurately evaluating the potential efficacy of rumen buffer materials in vitro. AUC appears to be the most robust and sensitive measure of buffering. It is evident that HCl-based titration methodologies overestimate the effectiveness of SB. The data also highlight the significant variation in the buffering capacity of CMA materials available commercially, likely due to their diversity of origin as well as their processing.

## Figures and Tables

**Table 1:** Compositional characterisation of calcium carbonate buffer materials.

Buffer	% Solids (*w*/*w*)	% Inorganic	% Organic	% Ca^2+^ *w*/*w*	% Mg^2+^ *w*/*w*
CMA Glac	99.88 ± 0.01 ^a^	96.65 ± 0.03 ^d^	3.35 ± 0.03 ^b^	29.49 ± 0.20 ^d^	5.67 ± 0.15 ^a^
CMA Cal 1	99.68 ± 0.05 ^b^	98.04 ± 0.08 ^c^	1.96 ± 0.08 ^c^	32.31 ± 0.20 ^c^	4.23 ± 0.05 ^b^
CMA Cal 2	99.52 ± 0.02 ^c^	96.37 ± 0.08 ^e^	3.63 ± 0.08 ^a^	32.21 ± 0.14 ^c^	2.40 ± 0.02 ^c^
CMA Cal 3	99.86 ± 0.03 ^a^	99.09 ± 0.03 ^b^	0.92 ± 0.03 ^d^	37.42 ± 0.39 ^a^	0.11 ± 0.01 ^d^
Calc Carb	99.95 ± 0.05 ^a^	99.56 ± 0.02 ^a^	0.45 ± 0.02 ^e^	35.95 ± 0.12 ^b^	0.13 ± 0.01 ^d^

Data are means ± standard error, n = 3. Statistically significant differences identified with superscript letters were determined using one-way ANOVA and Tukey’s post hoc test (*p* < 0.05).

**Table 2:** Particle size analysis of calcium carbonate buffer materials.

Buffer	Dx 10	Dx 25	Dx 50	Dx 90	Dx 95	Dx 99
CMA Glac	2.07 ± 0.05 ^cd^	5.14 ± 0.25 ^cd^	30.17 ± 4.96 ^b^	221.67 ± 3.57 ^b^	262.67 ± 2.28 ^c^	321 ± 1.29 ^c^
CMA Cal 1	5.87 ± 0.06 ^a^	20.4 ± 0 ^a^	92.27 ± 0.46 ^a^	254.67 ± 0.30 ^a^	287.33 ± 0.19 ^a^	333.67 ± 0.30 ^a^
CMA Cal 2	2.28 ± 0.02 ^c^	5.90 ± 0.03 ^c^	32.93 ± 0.27 ^b^	224.67 ± 0.30 ^b^	264.67 ± 0.30 ^bc^	322 ± 0 ^c^
CMA Cal 3	1.8 ± 0.01 ^d^	4.6 ± 0.01 ^d^	16 ± 0.1 ^c^	209 ± 0.30 ^c^	254 ± 0.80 ^d^	314 ± 1.60 ^d^
Calc Carb	3.21 ± 0.06 ^b^	7.70 ± 0.11 ^b^	26.63 ± 0.47 ^b^	221.67 ± 0.30 ^b^	270 ± 0.3 ^b^	325 ± 0.30 ^b^

Values are GMD (Geometric Mean Diameter) reported in µM. Dx 10 − Dx 99 = GMD of which 10–99% of sample mass has a GMD less than the recorded size. n = 3. Statistically significant differences identified with superscript letters were determined using one-way ANOVA and Tukey’s post hoc test (*p* < 0.05).

**Table 3:** Evaluation of the precision and uncertainty of measurement of static pH titration methodology over 2 h for the evaluation of rumen buffers.

Buffer	STAT Titration pH	Evaluation	Mean Vol HCl (mL)	Std	%CV	UoM (ml HCl)
CMA Glac	5.5	Between day	27.48	0.88	3.19	1.03
		Within day	27.99	0.54	1.94	
	6	Between day	23.73	0.79	3.34	0.99
		Within day	25.31	0.59	2.31	
CMA Cal 1	5.5	Between day	13.69	0.27	1.95	0.51
		Within day	13.24	0.43	3.27	
	6	Between day	11.78	0.75	6.37	0.93
		Within day	12.33	0.55	4.46	
CMA Cal 2	5.5	Between day	22.72	0.79	3.47	1.14
		Within day	22.31	0.82	3.70	
	6	Between day	19.37	1.83	9.44	2.09
		Within day	18.17	1.01	5.56	
CMA Cal 3	5.5	Between day	21.75	0.97	4.46	1.13
		Within day	21.98	1.29	5.89	
	6	Between day	17.27	1.04	6.00	1.06
		Within day	17.24	0.46	2.70	
Calc Carb	5.5	Between day	11.14	2.09	18.72	4.39
		Within day	13.43	3.86	28.74	
	6	Between day	12.49	1.89	15.16	2.55
		Within day	11.00	1.72	15.59	
SB 2X	5.5	Between day	57.60	0.61	1.10	0.97
		Within day	57.61	0.75	1.30	
	6	Between day	57.18	0.66	1.15	1.39
		Within day	57.19	1.22	2.13	

UoM = uncertainty of measurement, n = 5 replicates (within day) or 5 days (between day), %CV = coefficient of variation, Std = standard deviation, CMA = calcareous marine algae, Calc Carb = calcium carbonate/limestone, SB = sodium bicarbonate.

**Table 4:** Acid binding and buffer capacity of rumen buffer materials at pH 5.5 (subacute acidosis) over 2 h and 8 h using static pH titration methodology.

Time	Buffer	n	Initial pH	Mean HCl Total (mL)	%CV	ABC Meq/Kg	BUF
2 h	CMA Glac	5	10.20	27.48	3.19	11,194 ^d^	2526 ^de^
	CMA Cal 1		9.64	13.69	1.95	5296 ^f^	1285 ^g^
	CMA Cal 2		9.95	22.72	3.47	8924 ^e^	2205 ^ef^
	CMA Cal 3		9.69	21.75	4.46	8699 ^e^	2077 ^f^
	Calc Carb		9.59	11.14	18.72	5372 ^f^	1366 ^g^
	SB 2X		8.18	57.60	1.30	23,043 ^a^	8598 ^a^
8 h	CMA Glac	5	10.20	45.13	1.53	17,979 ^b^	3878 ^b^
	CMA Cal 1		9.63	28.56	7.55	11,089 ^d^	2693 ^d^
	CMA Cal 2		9.92	38.16	4.20	14,930 ^c^	3657 ^bc^
	CMA Cal 3		9.73	36.62	2.20	14,650 ^c^	3468 ^c^
	Calc Carb		9.57	28.89	3.93	11,222 ^d^	2794 ^d^
	SB 2X		8.16	60.32	0.15	24,127 ^a^	9060 ^a^

%CV = coefficient of variation; ABC = acid binding capacity; BUF = buffering capacity; n= replicates; Statistically significant differences identified with superscript letters were determined using one-way ANOVA and Tukey’s post hoc test (*p* < 0.05).

**Table 5:** ANOVA of fixed HCl acid load titration of different buffer materials using additional rumen relevant buffering parameters to assess titration curves.

Variable	AUC (mmol H^+^.s)	dpH/dt	Time (h) pH > Threshold
Buffer Material	*p* < 0.001	*p* < 0.001	*p* < 0.001
Threshold pH	*p* < 0.001	*p* = 0.966	*p* < 0.001
Duration of test	*p* < 0.001	*p* < 0.001	*p* = 1.000
			
Buffer Material			
SB 2X	34.13 ± 1.06 ^a^	1.84 ± 0.01 ^a^	9.50 ± 0.00 ^a^
CMA Glac 1X	24.37 ± 0.29 ^b^	5.15 ± 0.06 ^c^	7.73 ± 0.04 ^b^
CMA Cal 1 1X	13.21 ± 0.74 ^d^	7.26 ± 0.10 ^e^	5.55 ± 0.10 ^e^
CMA Cal 2 1X	20.83 ± 0.68 ^c^	6.32 ± 0.27 ^d^	7.08 ± 0.09 ^c^
CMA Cal 3 1X	22.08 ± 0.68 ^c^	4.74 ± 0.08 ^b^	6.70 ± 0.00 ^d^
Calc Carb 1X	7.73 ± 1.13 ^e^	7.15 ± 0.11 ^de^	3.78 ± 0.10 ^f^
			
Threshold pH			
5.5	35.84 ± 0.78 ^a^	5.38 ± 0.09	6.94 ± 0.05 ^a^
5.8	16.13 ± 0.31 ^b^	5.42 ± 0.09	6.73 ± 0.04 ^b^
6.0	9.20 ± 0.53 ^c^	5.42 ± 0.09	6.50 ± 0.06 ^c^
			
Duration (h)			
2	11.22 ± 0.40 ^a^	4.99 ± 0.09 ^a^	-
8	29.56 ± 0.52 ^b^	5.83 ± 0.05 ^b^	-
			
Interactions			
Buffer × Duration	*p* < 0.001	*p* < 0.001	*p* = 1.000
Buffer × pH	*p* < 0.001	*p* = 1.000	*p* = 0.301
pH × Duration	*p* < 0.001	*p* = 1.000	*p* = 1.000
Buffer × pH × Duration	*p* < 0.001	*p* = 0.966	*p* = 1.000

1X = standard dose; 2X = double standard dose; AUC = area under the curve expressed as mmol of hydrogen ion × second, ± standard error; dpH/dt = average rate of change of pH with time in seconds, CMA = calcareous marine algae, n = 5; pairwise comparison using Tukey’s post hoc test, *p* < 0.05.; superscript letters denote statistically significant differences.

**Table 6:** ANOVA of 3 h acidotic diet simulation for the different feed buffer materials.

Variable	AUC (mmol H^+^.s)	dpH/dt	Time(h) pH > Threshold
Buffer Material	*p* < 0.001	*p* < 0.001	*p* < 0.001
Threshold pH	*p* < 0.001	*p* = 1.000	*p* < 0.001
Duration of test	*p* < 0.001	*p* = 0.001	*p* = 1.000
			
Buffer Material			
SB 2X	9.85 ± 0.41 ^a^	0.78 ± 0.08 ^ab^	3.13 ± 0.09 ^a^
CMA Glac 1X	9.58 ± 0.23 ^a^	0.44 ± 0.01 ^c^	3.09 ± 0.05 ^a^
CMA Cal 1 1X	5.73 ± 0.16 ^d^	0.57 ± 0.02 ^b^	2.01 ± 0.04 ^c^
CMA Cal 2 1X	6.77 ± 0.21 ^c^	0.73 ± 0.07 ^ab^	2.39 ± 0.05 ^b^
CMA Cal 3 1X	7.94 ± 0.11 ^b^	0.81 ± 0.03 ^a^	2.90 ± 0.03 ^a^
Calc Carb 1X	5.74 ± 0.21 ^d^	0.66 ± 0.04 ^b^	2.01 ± 0.07 ^c^
			
Threshold pH			
5.5	18.69 ± 0.26 ^a^	0.66 ± 0.04	4.22 ± 0.05 ^a^
5.8	3.69 ± 0.11 ^b^	0.66 ± 0.04	2.34 ± 0.03 ^b^
6.0	0.43 ± 0.07 ^c^	0.66 ± 0.04	1.20 ± 0.03 ^c^
			
Duration (h)			
2	6.12 ± 0.09 ^a^	0.76 ± 0.03 ^a^	-
8	9.09 ± 0.18 ^b^	0.57 ± 0.03 ^b^	-
			
Interactions			
Buffer × Duration	*p* = 0.004	*p* = 0.986	-
Buffer × pH	*p* < 0.001	*p* = 1.000	*p* < 0.001
pH × Duration	*p* < 0.001	*p* = 1.000	-
Buffer × pH × Duration	*p* = 0.079	*p* = 1.000	-

AUC = area under the curve; dpH/dt = rate of change of pH with time (s); CMA = calcareous marine algae; - = not tested/relevant to test, n = 5; pairwise comparison completed using Tukey’s post hoc test, *p* < 0.05; different superscript letters denote statistically significant differences

**Table 7:** Calculated AUC from in vivo rumen acidosis trial (threshold pH = 5.8).

Buffer	AUC (mmol H^+^.s)
	0–3 h
CMA Glac	14.1 ± 1.12 ^a^
SB 2X	14.3 ± 0.36 ^a^
Calc Carb	9.65 ± 1.73 ^b^

n = 4. Pairwise comparison completed using Tukey’s post hoc test, *p* < 0.05; different superscript letters denote statistically significant differences.

## Data Availability

The original contributions presented in the study are included in the article/Supplementary Materials; further inquiries can be directed to the corresponding author.

## Supplementary Materials

The following supporting information can be downloaded at: https://www.mdpi.com/article/10.3390/ani14162333/s1, Figure S1: Rumen pH profile from in vivo sub-acute rumen acidosis trial comparing SB and CMA Glac from Neville et al. [15]; Figure S2: Rumen total VFA profile of the un-buffered control for the in vivo sub-acute rumen acidosis trial from Neville et al. [15]; Table S1. Calculation of mmol of acetic acid addition to 3 h in vitro acidosis simulation using VFA concentration produced over 180 min from in vivo rumen acidotic diet trial using cannulated dairy cows (raw data generated by Neville et al. [15]); Figure S3: A comparison of the titration curves generated for each buffer material tested using the fixed HCl acid load methodology with respect to an optimal rumen threshold pH of 6.0; Figure S4: An illustration of the features of the titration curves that are summarised and described by the calculation of parameters dpH/dt and AUC generated for each buffer material tested using the fixed HCl acid load and acidotic diet methodologies. The red line indicates the pH threshold of 6.0; Figure S5: A comparison of the titration curves generated for each buffer material tested using the acidotic diet simulation, with a rumen threshold pH of 5.8 indicated by the red line.

## Abbreviations

HCl—Hydrochloric acid; ABC—acid binding capacity; VFAs—volatile fatty acids; BUF—buffering capacity; BC—buffer capacity, BVI—buffer value index; CMA—calcareous marine algae; h—hour; min—minute; %CV—percentage coefficient of variation; CMA Glac—CMA Glaciale; CMA Cal (1 or 2 or 3)—Lithothamnium Calcareum; SB—sodium bicarbonate; mM—Millimolar; ANOVA—analysis of variance; CO_2_—carbon dioxide.
