# Supplementary material for: Evaluation and Development of Analytical Procedures to Assess Buffering Capacity of Carbonate Ruminant Feed Buffers
Source: Animals (Basel). 2024 Aug 13;14(16):2333. doi: 10.3390/ani14162333 (PMC11350906; doi:10.3390/ani14162333)
Supplement: Supplementary file 1 [file animals-14-02333-s001.zip › animals-3132284-supplementary.pdf]

Supplementary Material

Article

Evaluation and Development of Analytical Procedures to Assess Buffering Capacity of Carbonate Ruminant Feed Buffers

Patrick Quille [1], Tommy Higgins [2], Enda W. Neville [3], Katy Regan [3] and Shane O’Connell [1,2,*]

[1] Shannon Applied Biotechnology Centre, Munster Technological University Kerry, Clash, V92CX88 Tralee, Ireland
[2] Marigot Researh Centre, Sycamore Court, Clash, V92 N6C8 Tralee, Ireland
[3] Celtic Sea Minerals, Strand Farm, Currabinny, P43 NN62 Carrigaline, Ireland
* Correspondence: shane.oconnell@marigot.ie

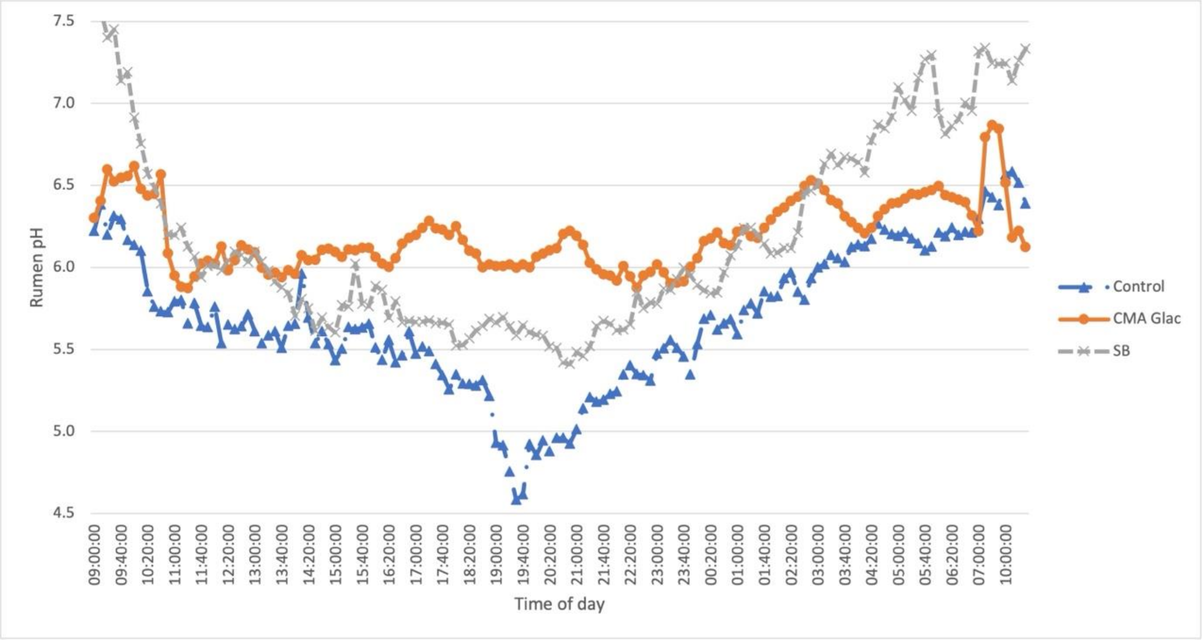

Figure S1: Rumen pH profile from *in vivo* sub acute rumen acidosis trial comparing SB 2X and CMA Glac from Neville et. al. [15]

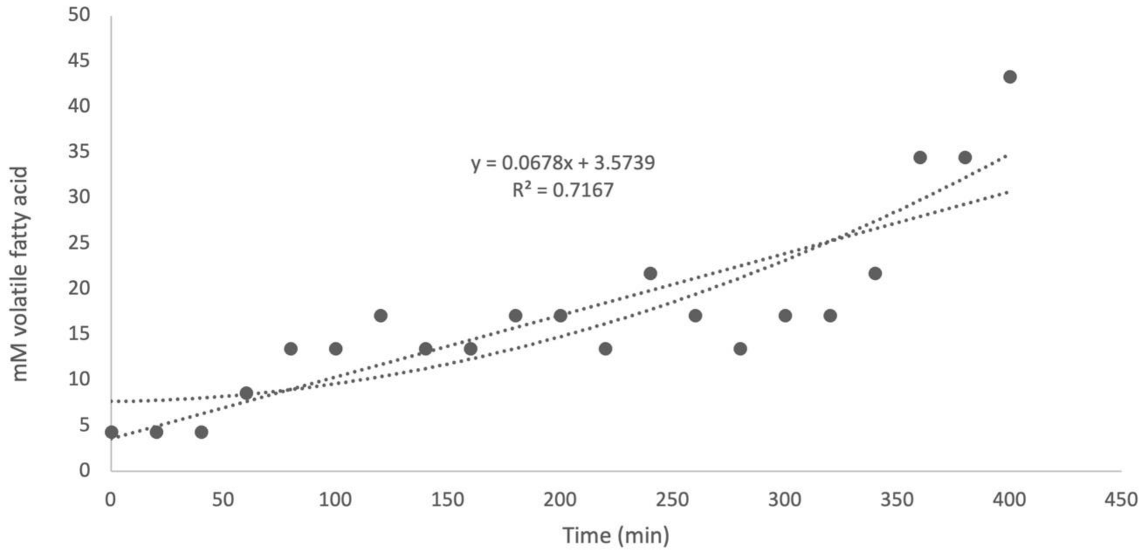

Figure S2: Rumen total VFA profile of the un-buffered control for the *in vivo* sub acute rumen acidosis trial from Neville et. al. [15]

Table S1. Calculation of mmol of acetic acid addition to 3 hour *in vitro* acidosis simulation using VFA concentration produced over 180 min from *in-vivo* rumen acidotic diet trial using cannulated dairy cows. (Raw data generated by Neville *et al.* [15]).

**Acid accumulation**	**Average of 180min**
mM acid/min	0.090
Addition mM per 25 min	2.250
Conversion to mmoles of acetic acid	0.337

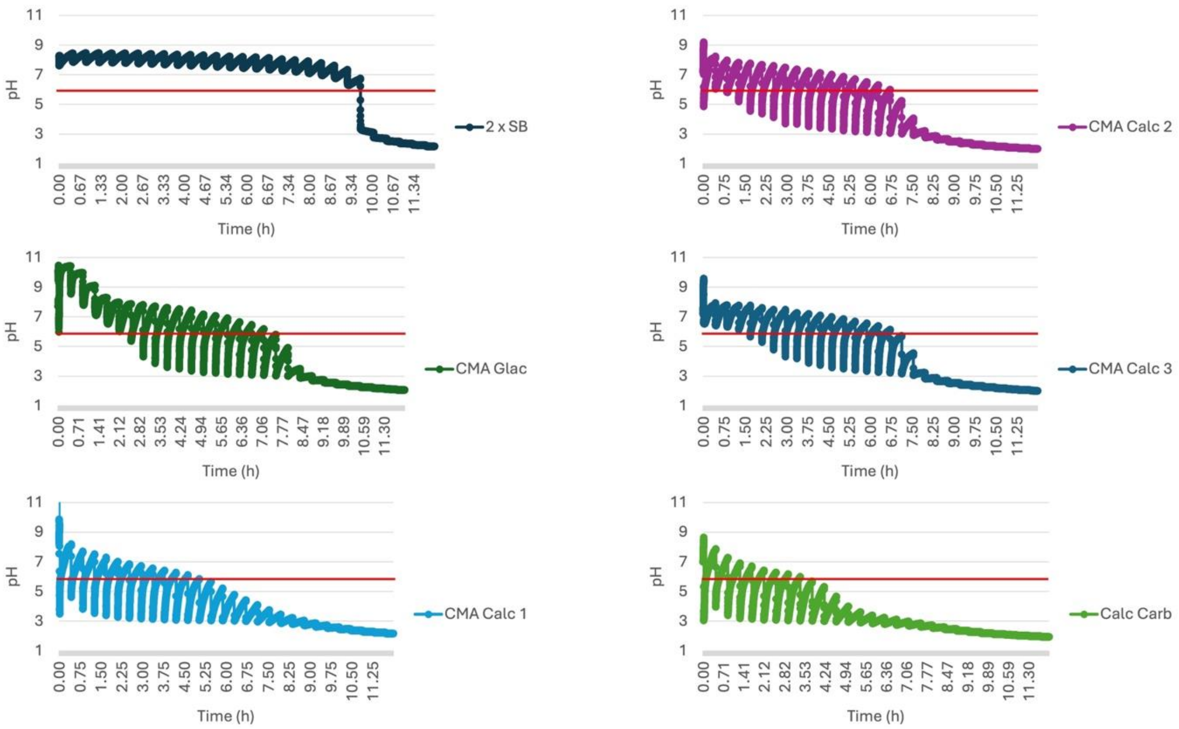

Figure S3: A comparison of the titration curves generated for each buffer material tested using the fixed HCl acid load methodology with respect to an optimal rumen threshold pH of 6.0.

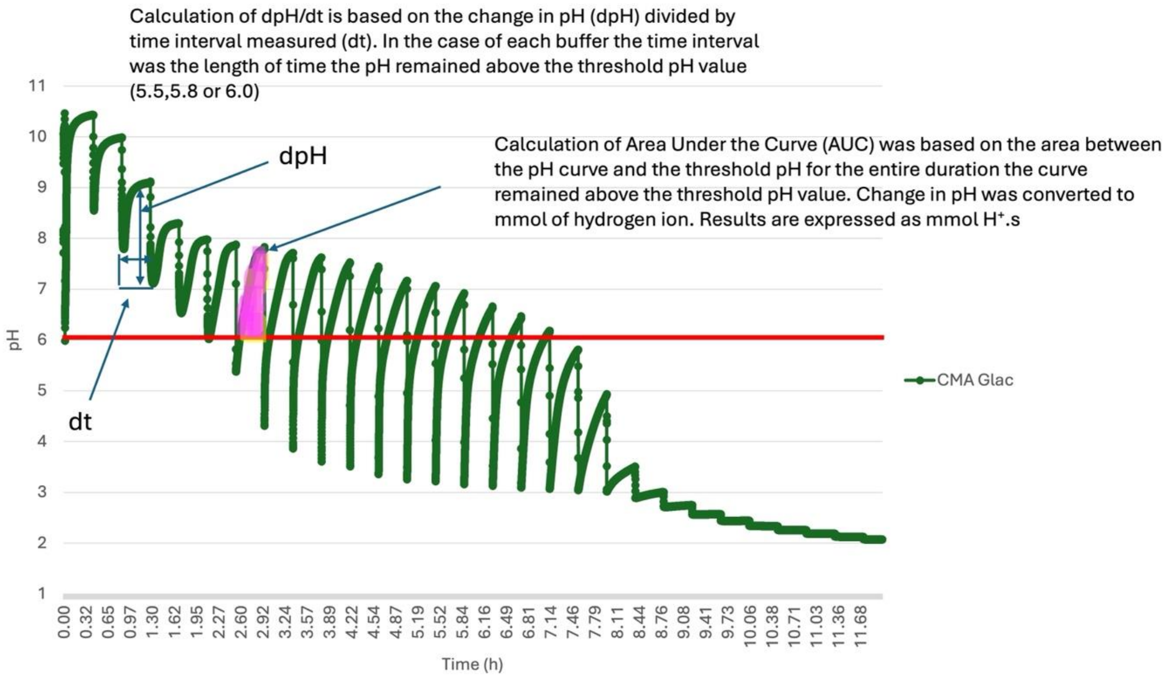

Figure S4: An illustration of the features of the titration curves that are summarise and described by the calculation of parameters dpH/dt and AUC generated for each buffer material tested using the fixed HCl acid load and acidotic diet methodologies. The red line indicates the pH threshold of 6.0

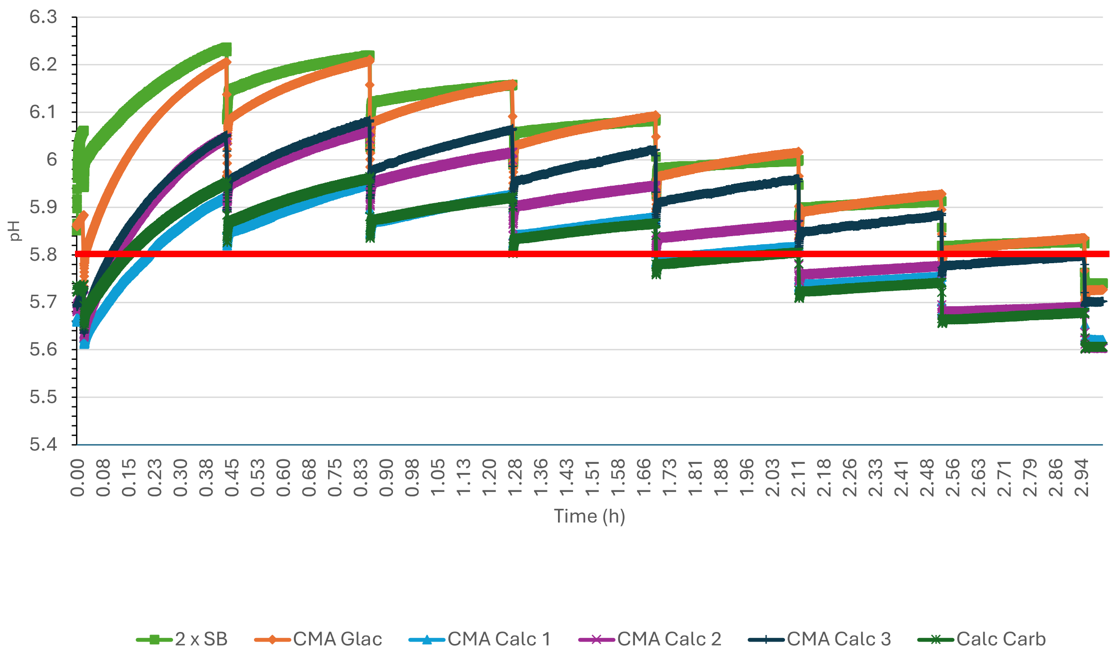

Figure S5: A comparison of the titration curves generated for each buffer material tested using the acidotic diet simulation with a rumen threshold pH of 5.8 indicated by the red line.
